# In Vitro Corrosion of SiC-Coated Anodized Ti Nano-Tubular Surfaces

**DOI:** 10.3390/jfb12030052

**Published:** 2021-09-16

**Authors:** Shu-Min Hsu, Chaker Fares, Xinyi Xia, Md Abu Jafar Rasel, Jacob Ketter, Samira Esteves Afonso Camargo, Md Amanul Haque, Fan Ren, Josephine F. Esquivel-Upshaw

**Affiliations:** 1 Department of Restorative Dental Sciences, Division of Prosthodontics, University of Florida College of Dentistry, Gainesville, FL 32610, USA; shuminhsu@ufl.edu (S.-M.H.); safonsocamargo@ufl.edu (S.E.A.C.); 2 Department of Chemical Engineering, University of Florida, Gainesville, FL 32610, USA; c.fares@ufl.edu (C.F.); xiaxinyi@ufl.edu (X.X.); fren@che.ufl.edu (F.R.); 3 Department of Mechanical Engineering, Penn State University, University Park, PA 16802, USA; mfr5667@psu.edu (M.A.J.R.); mah37@psu.edu (M.A.H.); 4 Gamry Instruments, Warminster, PA 18974, USA; JKetter@gamry.com

**Keywords:** corrosion, peri-implantitis, titanium implant, surface modification

## Abstract

Peri-implantitis leads to implant failure and decreases long-term survival and success rates of implant-supported prostheses. The pathogenesis of this disease is complex but implant corrosion is believed to be one of the many factors which contributes to progression of this disease. A nanostructured titanium dioxide layer was introduced using anodization to improve the functionality of dental implants. In the present study, we evaluated the corrosion performance of silicon carbide (SiC) on anodized titanium dioxide nanotubes (ATO) using plasma-enhanced chemical vapor deposition (PECVD). This was investigated through a potentiodynamic polarization test and bacterial incubation for 30 days. Scanning electron microscopy (SEM) and transmission electron microscopy (TEM) were used to analyze surface morphologies of non-coated and SiC-coated nanotubes. Energy dispersive X-ray (EDX) was used to analyze the surface composition. In conclusion, SiC-coated ATO exhibited improved corrosion resistance and holds promise as an implant coating material.

## 1. Introduction

Titanium and titanium alloy (Ti-6Al-4V) implants have been widely used to restore the function of missing teeth [1,2]. Several studies have demonstrated high survival and success rates for titanium and titanium alloys and the prostheses they support [2,3,4]. Titanium and titanium alloy implants exhibit relatively high strength, corrosion resistance, and biocompatibility [5,6,7,8,9]. Lekholm et al. reported a 92.6% overall implant survival rate after 10-years for Branemark implants on partially edentulous patients [2]. Pierre et al. showed an 82.94% long-term cumulative survival rate up to 16 years follow-up [10]. In spite of the reported high survival rate for these implants, biological (peri-implantitis) and/or technical (screw loosening and fracture) complications can lead to implant failure and decrease the success of implant-supported prostheses. The cumulative complication rate was 48.03% and the cumulative success rate was 51.97% for an observation period of up to 16 years [10].

Peri-implantitis is a multifactional process and is accompanied by tissue inflammation and loss of peri-implant bone [11,12]. Peri-implantitis is one of two pathologies classified under peri-implant disease. Dreyer et al. reported a systematic review on epidemiology and risks factors of peri-implantitis based on the publications from 1980 to 2016 [11]. The results showed the prevalence of peri-implantitis was up to 85.0% and the incidence was 43.9% in five years. Several pathogens/bacteria such as *Porphyromonas gingivalis* (*Pg*), *Prevotella intermedia* (*Pi*), *Tannerella forsythia* (*Tf*), and *Fusobacterium nucleatum* (*Fn*) were found to be highly associated with peri-implantitis [13,14]. Among these microorganisms, *P. gingivalis* accounts for the highest percentage of bacteria in biofilm formation on dental implants is capable of producing several virulence factors [15,16].

Implant corrosion is suspected to be one of the potential causes for implant failure [17]. Titanium or titanium alloys are very reactive to fluid or air. A titanium oxide layer is formed on the surface, where an interface between titanium and the oral environment is formed. This oxide layer is stable and protects titanium from corrosion. However, the oral environment is very hostile to dental materials in general, where these materials are exposed to masticatory forces, chemical, and bacteria environment, all of which can result in surface degradation [18,19,20,21]. Corrosion is a process where materials undergo degradation and release metallic ions into their surroundings. Mastication and loading can create cracks where the oxide layer can fracture [22]. Bacteria have been hypothesized to generate acid as a toxic byproduct, which lowers the pH of the environment and causes disruption of the oxide layer [23,24]. As the titanium oxide layer breaks down, the titanium implants become susceptible to corrosion.

Increasing long-term implant survival and success rates is important for predictability of implant supported restorations. This can be accomplished by materials that promote osseointegration, reduce the incidence and progression of peri-implantitis, have high strength, and are corrosion resistant. Surface modifications on titanium implants have been widely introduced in literature to improve osseointegration and/or reduce bacterial colonization [25,26,27]. Anodization is currently being used to produce a nanostructured titanium dioxide layer as a surface roughening modification technique because of the low cost, simplicity, controllability, and reproducibility of the process [28]. Brammer et al. showed that anodized titanium dioxide nanotubes exhibited greater bone-forming abilities as the diameter of nanotubes increased from 30 nm to 100 nm [29]. Peng et al. investigated the growth of *S. epidermidis* bacteria on titanium dioxide nanotubes and found bacteria decreased on nanotube surfaces compared with mechanically polished and acid-etched titanium sheets [30]. Aside from surface roughening modifications, surface coating is an alternative technique to improving physical properties of the surface. Fouda et al. showed that hydroxyapatite coated implants shorten the healing process compared with uncoated implants [31]. Das et al. demonstrated that silver-coated titanium oxide nanotubes have a higher antibacterial activity compared with only titanium oxide nanotubes [32].

Silicon carbide (SiC) coating has been used for biomedical applications due to this material’s high strength, corrosion resistance, lightweight, and biocompatibility [33,34,35,36,37,38]. The cytocompatibility of SiC coating was reported by Naji and Harmand [39]. SiC exhibited a better cytocompatibility for alveolar bone osteoblasts and gingival fibroblasts in comparison with titanium. Camargo et al. evaluated cytotoxicity of the SiC coating using human periodontal ligament fibroblasts and reported that SiC coating is biocompatible [38]. Hsu et al. demonstrated that SiC coating protects glass-ceramic veneers from corrosion [37]. Fares et al. demonstrated that SiC coating conformed to titanium implant surfaces and remained intact after torqueing into Poly(methyl methacrylate) blocks with similar human bone hardness [40].

In this pilot study, we determined (i) the ability of SiC coating to conform to titanium oxide nanotubes using plasma-enhanced chemical vapor deposition; (ii) the electrochemical stability of SiC-coated titanium nanotubes in NaCl solution compared with uncoated nanotubes; and (iii) the corrosion resistance of uncoated compared with SiC-coated titanium nanotubes to bacteria incubation after an extended period of time.

## 2. Materials and Methods

### 2.1. Samples Preparation

**Figure 1:**
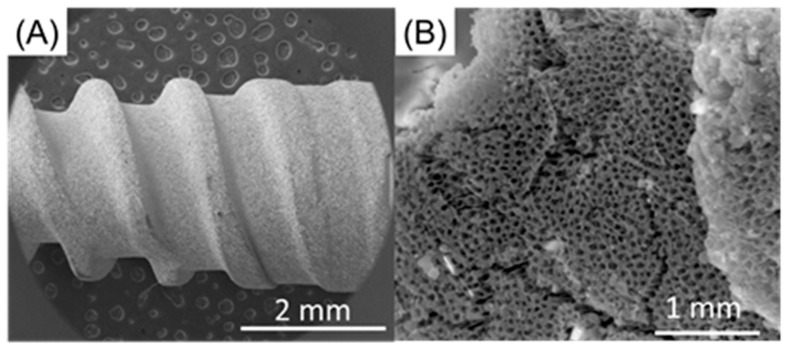
SEM images of anodized dental implants (**A**) low magnification (**B**) higher magnification.

Titanium oxide nanotubes were obtained through anodization, which was conducted in a two-electrode configuration with a DC power supply. The metallic titanium was oxidized where the titanium foil served as the anode and the graphite/platinum was the cathode. Anodized titanium dioxide (ATO) nanotubes on titanium foils were purchased in 100 nm and 150 nm size from InRedox (Longmont, CO, USA) and directly used in this experiment. The diameters of nanotubes on titanium foils that were chosen and used in this pilot experiment were based on nanotubes currently being applied to dental implant from Dentix Millenmium SRL (Giurgiu, Romania) (Figure 1).

A plasma-enhanced chemical vapor deposition (PECVD, PlasmaTherm 790, Saint Petersburg, FL, USA) was applied in this study. PECVD consists of a load lock, a parallel plate, and a showerhead. The coating deposition was conducted at 300 °C, where the silicon dioxide and silicon carbide (SiO_2_/SiC) coatings were coated on the ATO nanotubes. Silane and nitrous oxide were used for the SiO_2_ depositions as precursors. After SiO_2_ depostion, silicon-carbide was deposited using methane and silane as precursors. The chamber pressure was 1100 mTorr. Two thicknesses of coatings were applied in this study to determine if the coatings were able to conformably coat anodized titanium oxide nanotubes. They are (i) 12 nm SiO_2_ and 12 nm SiC, and (ii) 12 nm SiO_2_ and 30 nm SiC. The SiO_2_ was deposited on ATO initially and followed by SiC. We used 12 nm SiO_2_/SiC represented as 12 nm SiO_2_ and 12 nm SiC, and 30 nm SiO_2_/SiC represented as 12 nm SiO_2_ and 30 nm SiC in the following content.

### 2.2. Surface Characterization

Field-emission scanning electron microscopy (FEI Helios G4 PFIB CXe dual beam FIB, Thermo-Fisher Scientific Waltham, MA, USA) was utilized to examine the surface morphology of anodized titanium oxide nanotubes and SiO_2_/SiC-coated nanotubes. Energy Dispersive X-rays analysis was used to analyze the surface composition of the ATO nanotubes and SiO_2_/SiC-coated ATO nanotubes.

Scanning Electron Microscope (Helios Nanolab DualBeamTM, Thermo-Fisher Scientific Waltham, MA, USA) was used to analyze the cross-section of SiO_2_/SiC-coated ATO nanotubes. Electron transparent (nominally 100 nm thick) ATO nanotube coupons were prepared and lifted out using a Ga+ Focused Ion Beam (FIB). At first, a coupon was lifted out from the bulk sample and attached on a copper TEM grid which was further thinned down to 100 nm electron transparent sample using Ga+ FIB. Thinning down of the coupon involves a series of ion beam accelerating voltages and a wide range of current steps 21nA-72pA. The thickness of the sample was monitored at regular intervals during the thinning down process, and both accelerating voltage and currents were adjusted depending on the sample thickness. Energy dispersive spectroscopy (EDS) was performed inside a field emission 200 kV FEI Talos F200X TEM with 1.2 Å resolution.

### 2.3. Corrosion Tests

#### 2.3.1. Potentiodynamic Polarization Test

To evaluate corrosion behavior of the ATO nanotubes and SiO_2_/SiC-coated ATO nanotubes, a potentiodynamic polarization test was performed using a computer-controlled potentiostat (Gamry Interface 1010, Gamry Instruments, Warminster, PA, USA). The corrosion experiment was conducted in a three electrode flat cell with electroplaters tape used to minimize crevice formation. The ATO nanotubes and SiO_2_/SiC-coated ATO nanotubes were used as working electrode. The graphite was used as counter electrode. The saturated calomel electrode (SCE) was used as reference electrode. The working electrolyte was 3.5% NaCl solution.

#### 2.3.2. Bacterial Corrosion Test

ATO nanotubes and SiO_2_/SiC-coated ATO nanotubes were sterilized at 120 °C in an autoclave for 60 min. After sterilization, the samples were placed into individual sterile plates. There are three samples for each group. A biofilm of *Porphyromonas gingivalis* (FDC 381) was grown in Brucella blood agar plate supplemented with hemin and vitamin K (Hardy Diagnostics, Santa Maria, CA, USA). The axenic nature of the bacteria was assessed by Gram staining. The number of bacterial cells in the suspension was determined using Petroff-Hausser bacterial counting chamber. Bacteria were diluted in RTF to reach the final concentrations of 10^10^ cells/mL. ATO nanotubes and 12 nm SiO_2_/SiC-coated ATO nanotubes were placed in 1 mL of bacterial suspension individually in 24-well sterile plates in anaerobic chamber. The 24-well plates were maintained in anaerobic growth chamber for 30 days with respective fresh media replenishments for every two days. After 30 days, the biofilm was removed from the ATO nanotubes and SiO_2_/SiC-coated ATO nanotubes using a sonication for 5 min and examined under SEM.

## 3. Results

**Figure 2:**
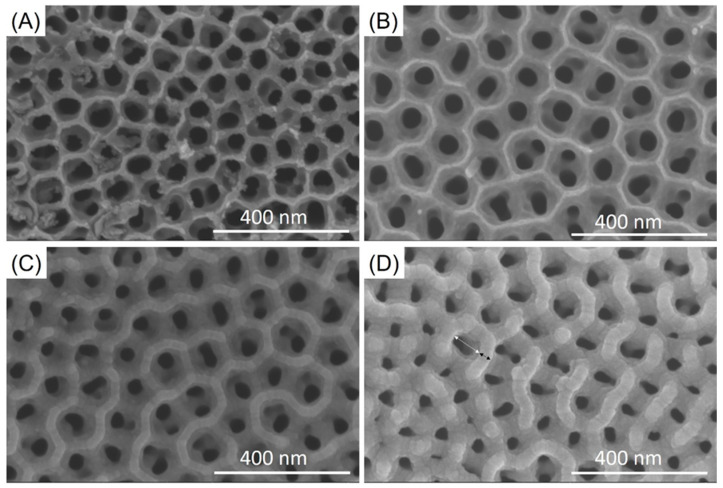
SEM images of (**A**) 100 nm ATO nanotubes, (**B**) 150 nm ATO nanotubes, (**C**) 12 nm SiO_2_/SiC/150 nm ATO nanotubes, (**D**) 30 nm SiO_2_/SiC/150 nm ATO nanotubes. The white arrow indicated the diameter of nanotubes and the black arrow indicated the ridge.

The nanotubes were fabricated using anodization. Figure 2 illustrates the ATO nano-tubes with 100 nm and 150 nm diameters from InRedox before SiO_2_/SiC coating and after SiO_2_/SiC coating. From the SEM results, the coatings demonstrated the ability to conform to the nanotube surface. The diameters of ATO nanotubes before coating and after coating were measured from SEM images and analyzed. The average diameter was 83 ± 10 nm with ridge 17 ± 6 nm for 100 nm ATO nanotubes, and 105 ± 30 nm with ridge 11 ± 11 nm for 150 nm ATO nanotubes (Table 1). After SiO_2_/SiC coating deposition, the diameter of ATO nanotubes and ridge of nanotubes were examined. Two different thicknesses of coating (12 nm SiO_2_/SiC and 30 nm SiO_2_/SiC) were applied to adjust the size of the nanotubes. The average diameter was 96 ± 12 nm with ridge 29 ± 2 nm for 12 nm SiO_2_/SiC-coated ATO nanotubes and was 85 ± 11 nm with ridge 41 ± 2 nm for 30 nm SiO_2_/SiC-coated ATO nanotubes.

**Figure 3:**
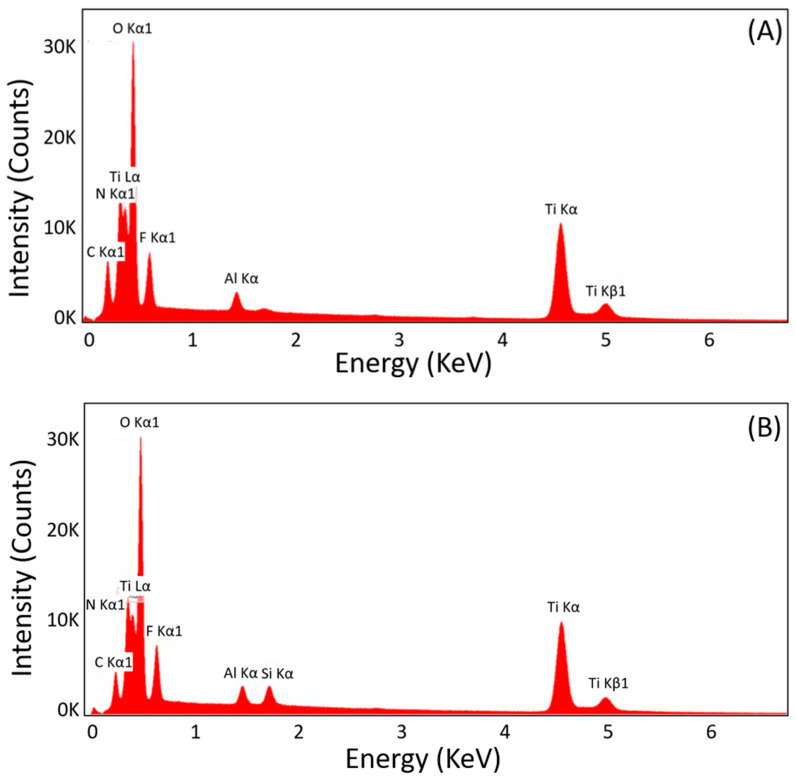
Representative EDX spectra of (**A**) non-coated ATO nanotubes and (**B**) SiO_2_/SiC-coated ATO nanotubes.

The ATO nanotubes and SiO_2_/SiC-coated ATO nanotubes were examined using energy-dispersive X-ray spectroscopy (EDX) analysis to determine the composition of the surface. The results were consistent among different diameters and thicknesses of ATO nanotubes. The representative EDX spectra are shown in Figure 3. Figure 3A shows the main elements as Ti, O, F, and Al from the non-coated ATO nanotubes. Figure 3B shows additional Si elements on ATO nanotubes after the SiO_2_/SiC coating was applied.

**Figure 4:**
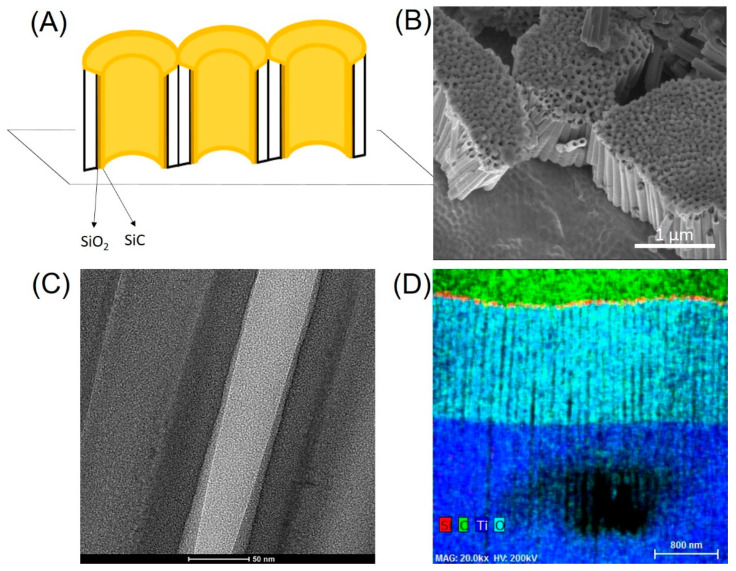
(**A**) SiO_2_/SiC coating on ATO nanotubes diagram, (**B**) SEM of bending SiO_2_/SiC/ATO nanotubes, (**C**) TEM cross-sectional images of SiO_2_/SiC/ATO nanotubes, and (**D**) EDX on cross-section TEM SiO_2_/SiC/ATO nanotubes.

Further investigation was conducted to determine the ability of SiO_2_/SiC coatings to conform to the internal surface of the ATO nanotubes. Figure 4A illustrates a schematic diagram of SiO_2_/SiC coating on ATO nanotubes, where the nanotubes were initially coated with SiO_2_ before the deposition of the SiC. The coating morphology was investigated with scanning electron microscopy (SEM) and transmission electron microscope (TEM). In order to achieve this, the titanium foil with coated ATO nanotubes was broken down by a mechanical force (i.e., scissor) into small pieces. This resulted in smaller clusters with a cross-sectional view of the ATO nanotubes arrays, as shown in Figure 4B. The ATO nanotubes were grown on the Ti foil substrates successfully using anodization, and coatings fully covered the surface of nanotubes. However, SEM microscopy did not show the internal surface of the nanotubes. For a higher magnification TEM study, a small section of the coated nanotubes were further processed into electron transparent lamella using the FIB technique. This allowed investigation of the internal surface of the nanotubes using TEM (relate Figure 4A–C). The results demonstrated the vertical alignment of SiO_2_/SiC-coated ATO nanotubes on the Ti foil substrate. The composition of cross-sectional ATO nanotubes using EDX (Figure 4D) revealed the presence Ti, O, Si, and C elements. The top region of the nanotubes showed Si and C elements mainly with Ti and O being the main elements towards the bottom portion of the nanotubes. The average diameter of these nanotubes is 51 ± 4 nm, obtained with higher magnification imaging. There is a difference between the diameter of ATO from the cross-sectional TEM measurements and the top SEM measurements (Table 1). The reason could be that the nanotubes were not cut off right at the middle positions and/or the diameter of ATO was larger at top portion and smaller towards the bottom portion of the ATO.

**Figure 5:**
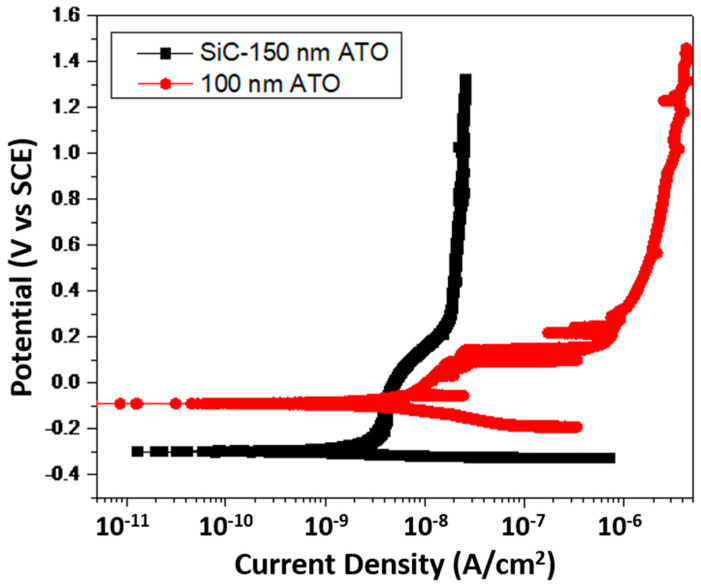
Polarization of ATO nanotubes and SiO_2_/SiC-coated ATO nanotubes.

The corrosion behavior of non-coated and coated ATO nanotubes was studied using potentiodynamic polarization test (Figure 5). The 100 nm ATO nanotubes and 12 nm SiO_2_/SiC-coated 150 nm ATO were used in this experiment. Figure 5 shows the anodic polarization curves of non-coated nanotubes and SiO_2_/SiC-coated nanotubes in 3.5% NaCl electrolyte. The passivation behavior was shown in the anodic polarization region for non-coated nanotubes and coated nanotubes. At the passivity region, the current density (i_0.1_) was 3.3 × 10^−7^ (A/cm^2^) for non-coated nanotubes and 7.5 × 10^−9^ (A/cm^2^) for SiO_2_/SiC-coated nanotubes. The anodic region of the potentiodynamic scans indicate less susceptibility to corrosion for SiO_2_/SiC-coated 150 nm ATO.

**Figure 6:**
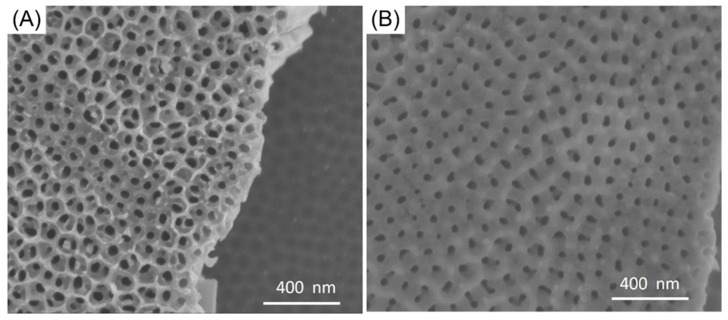
Corrosion of *P. gingivalis* on (**A**) 100 nm ATO nanotubes and (**B**) SiO_2_/SiC/150 nm ATO nanotubes.

The non-coated ATO nanotubes and SiO_2_/SiC-coated ATO nanotubes were evaluated for their corrosion resistance to *P. gingivalis* bacteria after a 30-day incubation period (Figure 6). The bacteria were removed from the surface after the 30-day incubation period. The samples were examined using SEM to determine the presence of corrosion. From SEM results, the surface demonstrated similar morphologies as before bacteria incubation (Figure 2A,C).

## 4. Discussion

Corrosion at the interface of dental implants is an important consideration as this phenomenon is believed to contribute to implant failure [41]. Studies have shown that metallic particles were found in the surrounding tissue as evidence of titanium corrosion. Failed implant surfaces were evaluated by Rodrigues and co-workers [42] and demonstrated corroded surfaces as evidenced by pitting, cracks, and discoloration, as these implants were exposed to masticatory forces, and an acidic environment from bacteria. Souza et al. evaluated titanium corrosion resistance in vitro in the presence of biofilm [24]. The results did not show localized corrosion after 48 h of biofilm growth. In addition, a report by Harada et al. showed no signs of corrosion or changes to surface morphology after using SEM on the titanium surfaces after *P. gingivalis* growth for seven days [43]. In this study, the ATO nanotubes and SiO_2_/SiC-coated ATO nanotubes specimens were incubated with *P. gingivalis* for 30 days. Based on these results, demonstration of localized corrosion or surface changes for non-coated ATO or SiO_2_/SiC-coated ATO nanotubes (Figure 2A,C and Figure 6) was inconclusive. However, the SiO_2_/SiC-coated ATO maintained a smooth surface after bacteria incubation compared with the non-coated. There is the possibility that a longer incubation time in bacteria, as what normally occurs intra-orally, can differentiate corrosion effects between coated and uncoated samples.

Corrosion behavior of titanium dioxide nanotubes has been widely evaluated using the potentiodynamic polarization test [44,45]. Titanium dioxide nanotubes showed enhanced corrosion resistance compared with mechanically polished titanium in artificial saliva [44]. Al-Saady’s study showed that corrosion behaviors of titanium dioxide nanotubes varied with the anodizing parameters [45]. The corrosion resistance improved as the applied voltage for anodization increased [45,46]. At the same time, the diameter of nanotubes was increased [44]. The area of nanotubes exposed to corrosive ions was increased. This may affect the electrochecmical corrosion behavior. The corrosion resistance was decreased when the diameter of titanium dioxide nanotubes was larger than 86 nm in a comparison of 22 nm to 59 nm diameters [44]. In our study, 100 nm ATO nanotubes were applied and the corrosion behavior was compared to SiO_2_/SiC-coated ATO nanotubes (Figure 5). ATO nanotubes possibly reacted with chloride ions in the electrolyte and became unstable [45], whereas SiO_2_/SiC-coated ATO nanotubes showed an improved corrosion resistance.

Surface coatings have been applied to ATO nanotubes to improve or enhance the desired function. However, previous studies have demonstrated that the surface morphology of ATO nanotubes may be altered because of the coatings. Roguska et al. reported an unexpected change to the surface, where partial nanotubes were closed by nanoparticles [47]. Perumal et al. showed the corrosion resistance of ATO nanotubes were improved by polyaniline polymer [48] but the pores of nanotubes were partially filled or disappeared after deposition of the polymer. Motola et al. deposited thin titanium dioxide coating on ATO nanotubes using atomic layer deposition to enhance cell growth [49]. The coating was deposited on nanotubes with 0.3 nm thickness successfully. However, the diameter of nanotubes decreased and some of them were clogged by titanium dioxide coatings when the coating thickness was increased to 8 nm. In this pilot study, SiO_2_/SiC coating conformably covered the ATO nanotubes without significant surface alteration (Figure 2 and Figure 4). The diameters of nanotubes can be adjusted by controlling the thickness of SiO_2_/SiC coatings (Table 1).

One limitation of this pilot study is the coating coverage inside of ATO nanotubes. From the SEM cross-section images (Figure 4B), thickened layers of the SiC coating were deposited on top of the nanotubes. From the TEM cross-section image and EDX (Figure 4C,D), a relatively higher content of Si and C on the top of the nanotubes is demonstrated and serves as evidence of the presence of coatings. Although the authors intended to examine the ATO nanotubes at cross-section (Figure 4A) using FIB, some of the nanotubes were difficult to visualize completely and may have affected the EDX results. Another limitation is although this pilot study demonstrates a promising approach in that SiO_2_/SiC coatings are capable to conform to the surface of ATO nanotubes utilizing PECVD, there is still a need to optimize the coating parameters and examine the coating coverage inside the nanotubes because the nanotubes can be a channel for the electrolyte, where the corrosion initiated. A third limitation is that the level of titanium ion released from long periods of bacterial incubation is still unknown. Results from bacterial inoculation of coated and non-coated nanotubes were inconclusive in this study. Future studies are recommended to consider quantifying the released titanium ions from bacteria or increasing incubation time.

## 5. Conclusions

In conclusion, a SiO_2_/SiC coating is capable of covering ATO nanotubes conformably. This pilot study showed an improved corrosion resistance on ATO nanotubes under 3.5% NaCl solutions. The SiO_2_/SiC-coated ATO maintained a smooth surface after bacterial incubation.

## Figures and Tables

**Table 1:** The results of diameters and ridges of ATO nanotubes before coatings and after coating deposition.

Size Parameters	100 nm ATO	150 nm ATO	12 nm SiO_2_/SiC 150 nm ATO	30 nm SiO_2_/SiC 150 nm ATO
Diameter (nm)	83 ± 10	105 ± 30	96 ± 12	85 ± 11
Ridge (nm)	17 ± 6	11 ± 11	29 ± 2	41 ± 2

## Data Availability

The data presented in this study are available on request from the corresponding author.

## References

[1] Adell R., Eriksson B., Lekholm U., Brånemark P.I., Jemt T. (1990). Long-term follow-up study of osseointegrated implants in the treatment of totally edentulous jaws. Int. J. Oral Maxillofac. Implant..

[2] Lekholm U.L.F., Gunne J., Henry P., Higuchi K., Lindén U., Bergström C., Van Steenberghe D. (1999). Survival of the Brånemark implant in partially edentulous jaws: A 10-year prospective multicenter study. Int. J. Oral Maxillofac. Implant..

[3] Schwartz-Arad D., Herzberg R., Levin L. (2005). Evaluation of long-term implant success. J. Periodontol..

[4] Mozzati M., Gallesio G., Del Fabbro M. (2015). Long-Term (9–12 Years) Outcomes of Titanium Implants With an Oxidized Surface: A Retrospective Investigation on 209 Implants. J. Oral Implant..

[5] de Assis S.L., Wolynec S., Costa I. (2006). Corrosion characterization of titanium alloys by electrochemical techniques. Electrochim. Acta.

[6] Mathew M.T., Kerwell S., Lundberg H.J., Sukotjo C., Mercuri L.G. (2014). Tribocorrosion and oral and maxillofacial surgical devices. Br. J. Oral Maxillofac. Surg..

[7] Hanawa T. (2011). A comprehensive review of techniques for biofunctionalization of titanium. J. Periodontal Implant. Sci..

[8] Cruz H.V., Souza J.C.M., Henriques M., Rocha L.A. (2011). Tribocorrosion and Bio-Tribocorrosion in the Oral Environment: The Case of Dental Implants.

[9] Apaza K., Tarce M., Benfatti C.A.M., Henriques B., Mathew M.T., Teughels W., Souza J.C.M. (2017). Synergistic interactions between corrosion and wear at titanium-based dental implant connections: A scoping review. J. Periodontal Res..

[10] Simonis P., Dufour T., Tenenbaum H. (2010). Long-term implant survival and success: A 10–16-year follow-up of non-submerged dental implants. Clin. Oral Implant. Res..

[11] Dreyer H., Grischke J., Tiede C., Eberhard J., Schweitzer A., Toikkanen S.E., Glöckner S., Krause G., Stiesch M. (2018). Epidemiology and risk factors of peri-implantitis: A systematic review. J. Periodontal Res..

[12] Cecchinato D., Parpaiola A., Lindhe J. (2012). A cross-sectional study on the prevalence of marginal bone loss among implant patients. Clin. Oral Implant. Res..

[13] De Waal Y.C.M., Eijsbouts H.V.L.C., Winkel E., Van Winkelhoff A. (2017). Microbial Characteristics of Peri-Implantitis: A Case-Control Study. J. Periodontol..

[14] Persson G.R., Renvert S. (2014). Cluster of Bacteria Associated with Peri-Implantitis. Clin. Implant. Dent. Relat. Res..

[15] Periasamy S., Kolenbrander P.E. (2009). Mutualistic biofilm communities develop with Porphyromonas gingivalis and initial, early, and late colonizers of enamel. J. Bacteriol..

[16] Tribble G.D., Kerr J.E., Wang B.-Y. (2013). Genetic diversity in the oral pathogen Porphyromonas gingivalis: Molecular mechanisms and biological consequences. Future Microbiol..

[17] Olmedo D.G., Tasat D.R., Duffó G., Guglielmotti M.B., Cabrini R.L. (2009). The issue of corrosion in dental implants: A review. Acta Odontol Lat..

[18] Rakic M., Grusovin M.G., Canullo L. (2016). The Microbiologic Profile Associated with Peri-Implantitis in Humans: A Systematic Review. Int. J. Oral Maxillofac. Implant..

[19] Canullo L., Peñarrocha-Oltra D., Covani U., Rossetti P.H.O. (2015). Microbiologic and Clinical Findings of Implants in Healthy Condition and with Peri-Implantitis. Int. J. Oral Maxillofac. Implant..

[20] Nikolopoulou F. (2006). Saliva and Dental Implants. Implant. Dent..

[21] Chaturvedi T. (2009). An overview of the corrosion aspect of dental implants (titanium and its alloys). Indian J. Dent. Res..

[22] Bhola R., Bhola S.M., Mishra B., Olson D.L. (2011). Corrosion in Titanium Dental Implanats/Prostheses—A Review. Trends Biomater Artif Organs.

[23] Mathew M.T., Barão V.A., Yuan J.C.C., Assunção W.G., Sukotjo C., Wimmer M.A. (2012). What is the role of lipopolysaccharide on the tribocorrosive behavior of titanium?. J. Mech. Behav. Biomed. Mater..

[24] Souza J.C., Ponthiaux P., Henriques M., Oliveira R., Teughels W., Celis J.-P., Rocha L.A. (2013). Corrosion behaviour of titanium in the presence of Streptococcus mutans. J. Dent..

[25] Xue T., Attarilar S., Liu S., Liu J., Song X., Li L., Zhao B., Tang Y. (2020). Surface Modification Techniques of Titanium and its Alloys to Functionally Optimize Their Biomedical Properties: Thematic Review. Front. Bioeng. Biotechnol..

[26] Jemat A., Ghazali M.J., Razali M., Otsuka Y. (2015). Surface Modifications and Their Effects on Titanium Dental Implants. BioMed Res. Int..

[27] Orapiriyakul W., Young P.S., Damiati L., Tsimbouri P.M. (2018). Antibacterial surface modification of titanium implants in orthopaedics. J. Tissue Eng..

[28] Izmir M., Ercan B. (2018). Anodization of titanium alloys for orthopedic applications. Front. Chem. Sci. Eng..

[29] Brammer K.S., Oh S., Cobb C.J., Bjursten L.M., Van Der Heyde H., Jin S. (2009). Improved bone-forming functionality on diameter-controlled TiO_2_ nanotube surface. Acta Biomater..

[30] Peng Z., Ni J., Zheng K., Shen Y., Wang X., He G., Jin S., Tang T. (2013). Dual effects and mechanism of TiO_2_ nanotube arrays in reducing bacterial colonization and enhancing C3H10T1/2 cell adhesion. Int. J. Nanomed..

[31] Fouda M.F.A., Nemat A., Gawish A., Baiuomy A.R. (2009). Does the Coating of Titanium Implants by Hydroxyapatite affect the Elaboration of Free Radicals. An Experimental Study. Aust. J. Basic Appl. Sci..

[32] Das K., Bose S., Bandyopadhyay A., Karandikar B., Gibbins B.L. (2008). Surface coatings for improvement of bone cell materials and antimicrobial activities of Ti implants. J. Biomed. Mater. Res. Part B Appl. Biomater..

[33] Brennan J.J., Prewo K.M. (1982). Silicon carbide fibre reinforced glass-ceramic matrix composites exhibiting high strength and toughness. J. Mater. Sci..

[34] Filardo G., Kon E., Tampieri A., Rodríguez R.C., Di Martino A., Fini M., Giavaresi G., Lelli M., Fernández J.M., Martini L. (2013). New Bio-Ceramization Processes Applied to Vegetable Hierarchical Structures for Bone Regeneration: An Experimental Model in Sheep. Tissue Eng. Part A.

[35] González P., Serra J., Liste S., Chiussi S., León B., Pérez-Amor M., Martínez-Fernández J., de Arellano-López A.R., Varela-Feria F.M. (2003). New biomorphic SiC ceramics coated with bioactive glass for biomedical applications. Biomaterials.

[36] Gryshkov O., Klyui N.I., Temchenko V.P., Kyselov V.S., Chatterjee A., Belyaev A.E., Lauterboeck L., Iarmolenko D., Glasmacher B. (2016). Porous biomorphic silicon carbide ceramics coated with hydroxyapatite as prospective materials for bone implants. Mater. Sci. Eng. C.

[37] Hsu S.-M., Ren F., Chen Z., Kim M., Fares C., Clark A.E., Neal D., Esquivel-Upshaw J.F. (2020). Novel Coating to Minimize Corrosion of Glass-Ceramics for Dental Applications. Materials.

[38] Camargo S.E.A., Mohiuddeen A.S., Fares C., Partain J.L., Carey P.H., Ren F., Hsu S.-M., Clark A.E., Esquivel-Upshaw J.F., Iv P.H.C. (2020). Anti-Bacterial Properties and Biocompatibility of Novel SiC Coating for Dental Ceramic. J. Funct. Biomater..

[39] Naji A., Harmand M.-F. (1991). Cytocompatibility of two coating materials, amorphous alumina and silicon carbide, using human differentiated cell cultures. Biomaterials.

[40] Fares C., Hsu S.-M., Xian M., Xia X., Ren F., Mecholsky J.J.J., Gonzaga L., Esquivel-Upshaw J. (2020). Demonstration of a SiC Protective Coating for Titanium Implants. Materials.

[41] Olmedo D., Fernández M.M., Guglielmotti M.B., Cabrini R.L. (2003). Macrophages related to dental implant failure. Implant. Dent..

[42] Rodrigues D.C., Valderrama P., Wilson J.T.G., Palmer K., Thomas A., Sridhar S., Adapalli A., Burbano M., Wadhwani C. (2013). Titanium Corrosion Mechanisms in the Oral Environment: A Retrieval Study. Materials.

[43] Harada R., Kokubu E., Kinoshita H., Yoshinari M., Ishihara K., Kawada E., Takemoto S. (2018). Corrosion behavior of titanium in response to sulfides produced by Porphyromonas gingivalis. Dent. Mater..

[44] Liu C., Wang Y., Wang M., Huang W., Chu P.K. (2011). Electrochemical stability of TiO2 nanotubes with different diameters in artificial saliva. Surf. Coat. Technol..

[45] Al-Saady F.A., Rushdi S.A., Abbar A.H. (2020). Improvement the Corrosion Behavior of Titanium by Nanotubular Oxide in a simulated saliva solution. IOP Conf. Ser. Mater. Sci. Eng..

[46] Yang G., Ma D., Liu L., Rong J., Yu X. (2017). Electrochemical Behavior Analyses of Anodic Oxide Film Obtained on TA2 Pure Titanium in Sulfuric Acid Electrolyte. Chem. Eng. Trans..

[47] Roguska A., Belcarz A., Pisarek M., Ginalska G., Lewandowska M. (2015). TiO_2_ nanotube composite layers as delivery system for ZnO and Ag nanoparticles—An unexpected overdose effect decreasing their antibacterial efficacy. Mater. Sci. Eng. C.

[48] Perumal A., Kanumuri R., Rayala S.K., Nallaiyan R. (2020). Fabrication of bioactive corrosion-resistant polyaniline/TiO_2_ nanotubes nanocomposite and their application in orthopedics. J. Mater. Sci..

[49] Motola M., Capek J., Zazpe R., Bacova J., Hromadko L., Bruckova L., Ng S., Handl J., Spotz Z., Knotek P. (2020). Thin TiO_2_ Coatings by ALD Enhance the Cell Growth on TiO_2_ Nanotubular and Flat Substrates. ACS Appl. Bio Mater..

